# A Novel Virus-Like Particle Based Vaccine Platform Displaying the Placental Malaria Antigen VAR2CSA

**DOI:** 10.1371/journal.pone.0143071

**Published:** 2015-11-23

**Authors:** Susan Thrane, Christoph M. Janitzek, Mette Ø. Agerbæk, Sisse B. Ditlev, Mafalda Resende, Morten A. Nielsen, Thor G. Theander, Ali Salanti, Adam F. Sander

**Affiliations:** 1 Centre for Medical Parasitology at the Department of Immunology and Microbiology, University of Copenhagen, Copenhagen, Denmark; 2 Department of Infectious Diseases, Copenhagen University Hospital, Copenhagen, Denmark; Ehime University, JAPAN

## Abstract

Placental malaria caused by *Plasmodium falciparum* is a major cause of mortality and severe morbidity. Clinical testing of a soluble protein-based vaccine containing the parasite ligand, VAR2CSA, has been initiated. VAR2CSA binds to the human receptor chondroitin sulphate A (CSA) and is responsible for sequestration of *Plasmodium falciparum* infected erythrocytes in the placenta. It is imperative that a vaccine against malaria in pregnancy, if administered to women before they become pregnant, can induce a strong and long lasting immune response. While most soluble protein-based vaccines have failed during clinical testing, virus-like particle (VLP) based vaccines (*e*.*g*., the licensed human papillomavirus vaccines) have demonstrated high efficacy, suggesting that the spatial assembly of the vaccine antigen is a critical parameter for inducing an optimal long-lasting protective immune response. We have developed a VLP vaccine display platform by identifying regions of the HPV16 L1 coat protein where a biotin acceptor site (AviTag^TM^) can be inserted without compromising VLP-assembly. Subsequent biotinylation of Avi-L1 VLPs allow us to anchor monovalent streptavidin (mSA)-fused proteins to the biotin, thereby obtaining a dense and repetitive VLP-display of the vaccine antigen. The mSA-VAR2CSA antigen was delivered on the Avi-L1 VLP platform and tested in C57BL/6 mice in comparison to two soluble protein-based vaccines consisting of naked VAR2CSA and mSA-VAR2CSA. The mSA-VAR2CSA Avi-L1 VLP and soluble mSA-VAR2CSA vaccines induced higher antibody titers than the soluble naked VAR2CSA vaccine after three immunizations. The VAR2CSA Avi-L1 VLP vaccine induced statistically significantly higher endpoint titres compared to the soluble mSA-VAR2CSA vaccine, after 1^st^ and 2^nd^ immunization; however, this difference was not statistically significant after 3^rd^ immunization. Importantly, the VLP-VAR2CSA induced antibodies were functional in inhibiting the binding of parasites to CSA. This study demonstrates that the described Avi-L1 VLP-platform may serve as a versatile system for facilitating optimal VLP-display of large and complex vaccine antigens.

## Introduction

Malaria caused by *Plasmodium falciparum* has severe consequences for hundreds of millions of individuals infected every year [1]. Pregnant women are at risk of developing placental malaria (PM), which is characterized by the binding of infected erythrocytes (IE) to the receptor chondroitin sulphate A (CSA) in the placenta. The accumulation of IEs in the placenta causes maternal anemia and fetal growth retardation [2–5]. The parasite ligand, VAR2CSA, belongs to the family of *Plasmodium falciparum* erythrocyte membrane protein 1 (PfEMP1) adhesins and is responsible for sequestration of IE [6–8]. In areas where malaria is endemic, women acquire protective antibodies as a function of parity [9] and these antibodies prevent sequestration of IE in the placenta by blocking the interaction between VAR2CSA and placental CSA [10]. Although these findings support the feasibility of developing a VAR2CSA-based PM vaccine, it should be noted that such a vaccine would have to be administered prior to conception, ideally to pre-puberty girls. Thus, efficient induction of immunological memory is required. Currently, two PM vaccines are in clinical development, and both are adjuvanted soluble protein-based vaccines consisting of a VAR2CSA recombinant protein, corresponding to the CSA-binding region of VAR2CSA. The immunogenicity of soluble protein-based vaccines is, however, generally low compared to that of full pathogen-based vaccines *e*.*g*. inactivated or live attenuated virus [11]. Vaccine studies have compensated for this by applying higher antigen doses, administering booster vaccinations and co-administrating adjuvants [12]. Even so, it has generally not been possible to induce potent and long-lived immune responses against soluble proteins, a sad fact exemplified by the long list of soluble protein-based vaccines that have failed in clinical efficacy trials [12]. By contrast, virus-like particles (VLPs) are known to induce remarkably rapid, strong and long-lasting antibody responses [11,13]. This is exemplified by data indicating that a single dose of the VLP-based human papillomavirus (HPV) vaccine (Cervarix), consisting of HPV L1 VLPs, induce serotype specific protection for at least 48 months [14]. HPV L1 VLPs are non-infectious protein assemblies consisting of the L1 major capsid protein. These viral structural proteins self-assemble during recombinant expression into supra-molecular complexes with structural characteristics similar to the parental virus, intrinsically rendering them highly immunogenic and thus combining many of the advantages of full pathogen-based vaccines and soluble protein-based vaccines into one system [15]. In addition to being effective vaccines against the virus from which they are derived, VLPs have also been used to present foreign epitopes to the immune system. This can be achieved either by genetic fusion of heterologous epitopes into the viral coat protein or by chemical conjugation to pre-assembled VLPs [16,17]. In either case, it is a requirement that the vaccine immunogen is presented at high density and in a consistent orientation in order to achieve optimal epitope spacing and mimic the repetitive, multivalent epitope-display of a virus [18,19]. Presenting a complex antigen (*e*.*g*. the VAR2CSA immunogen, 66kDa) on a VLP poses a significant biotechnological challenge, since it cannot be incorporated into the viral coat protein without compromising VLP assembly. Due to the size of VAR2CSA, it is likewise difficult to control the orientation and density of the coupled antigen using a traditional chemical conjugation approach (*e*.*g*. using hetero bi-functional cross-linkers).

In this study, we aimed to overcome these challenges by producing a genetically engineered HPV16 L1 VLP, which upon insertion of a biotin acceptor sequence (AviTag^TM^) in the L1 coding sequence, can be site-specifically biotinylated and used to present monovalent streptavidin (mSA)-fused antigens in an orderly manner. The immunogenicity of an Avi-L1 VLP-based VAR2CSA vaccine was tested against that of soluble VAR2CSA in C57BL/6 mice, and the obtained data serve as proof of concept for the Avi-L1 VLP platform, which may represent a versatile system for facilitating VLP-display of large vaccine antigens.

## Materials and Methods

### Design of chimeric HPV16 Avi-L1 coat proteins

The chimeric HPV16 Avi-L1 gene sequences were constructed by insertion of the biotin acceptor sequence (AviTag^TM^), **GLNDIFEAQKIEWHE,** into the DE- (aa pos. 134–137), FG- (aa pos. 278–285), HI- (aa pos. 350–351) or H4-βJ coil (aa pos. 429–430) after having deleted any intervening amino acids (HPV16 L1 accession number DQ155283.1). Gene sequences were further modified to contain an EcoRV restriction site followed by a polyhedrin promotor sequence at the N-terminus and a stop-codon followed by a NotI restriction site at the C-terminus. The synthetic gene sequences were finally codon-optimized for recombinant expression in Trichoplusia ni cells and synthesized by Geneart (Life Technologies).

### Expression and purification of chimeric HPV16 Avi-L1- VLPs

The HPV16 Avi-L1 gene fragments were cloned into the EcoRV/NotI sites of the pAcGP67A vector (BD Biosciences) deleting the gp67 secretion signal sequence. To generate recombinant virus particles, linearized BakPak viral DNA (BD Biosciences) was co-transfected with pAcGP67A/Avi-L1 into Sf9 insect cells using Lipofectamine 2000 10 Reagent (Invitrogen, 11668–019) and incubated at 28°C for 3–5 days. Recombinant Baculovirus was harvested from the supernatant and used to generate a high-titer virus stock, which was used for infection of High-Five insect cells. Infected High-Five cells were incubated for 48 hours at 28°C with shaking. *In vitro* maturation and purification of HPV16 Avi-L1 VLPs were performed as previously described [20,21]. In brief, cell lysates were harvested and VLPs were allowed to mature in maturation-buffer (0.5% Triton-X-100, 0.1% Benzonase®Nuclease (Sigma-Aldrich), 25 mM (NH_4_)_2_SO_4_ and 4mM MgCl_2_) for 18h at 37°C. Matured VLPs were subsequently purified by ultracentrifugation through an Optiprep^TM^ step gradient (27%/33%/39%) as previously described [22]. VLPs were dialyzed in PBS (0.02% PS80) and incubated for 30 min at 30°C with biotin and Biotin ligase (BirA) according to instructions from Avidity (Aurora, CO). Excess biotin was removed by dialysis in PBS (0.32 M NaCl, 0.02% PS80) and protein concentration was determined using BCA analysis. Collected ultracentrifugation fractions were analyzed with NuPAGE® Bis-Tris Protein gels (Life Technologies) or blotted onto a nitrocellulose membrane (GE-Healthcare, RPN203E) for detection of L1 or biotin with CamVir-1 (AbD Serotec, Bio-Rad, 7135–2804) or Streptavidin—HRP (Life Technologies, 43–4323), respectively. Densitometric analysis of SDS-PAGE gels was done using ImageJ.

### Electron microscopy–Negative staining

An aliquot of diluted VLPs was adsorbed to 200-mesh mica carbon-coated grids and negatively stained with 2% phosphotungstic acid (pH = 7.0). The sample was examined with a CM 100 BioTWIN electron microscope (Phillips, Amsterdam) at an accelerating voltage of 80 kV. Photographic records were performed on an Olympus Veleta camera. Particle sizes were estimated using ImageJ.

### Particle size measurement by dynamic light scattering (DLS)

The hydrodynamic diameter (referred to as size) of the VLP was measured using a particle analyzer, DynaPro NanoStar (WYATT Technology), equipped with a 658 nm laser. VLP samples were diluted to 0.2 mg/mL with PBS (0.32 M NaCl, 0.02% PS80) and 70 μl of each VLP sample was loaded into a disposable low volume cuvette and mounted into the DLS chamber. After 1 min equilibration, the size distribution was obtained by DLS measurement at 25°C. Each sample was measured 2 times with 20 runs in each measurement. The most predominant average sizes of particles in the population were calculated from the measurements and recorded together with the % polydispersity (%Pd).

### Expression and purification of VAR2CSA and mSA-VAR2CSA

The chimeric mSA-VAR2CSA gene sequence was designed with a small Gly-Gly-Ser linker separating the monomeric Streptavidin (mSA) [GenBank: 4JNJ_A] from the ID1-ID2a domains of VAR2CSA [FCR3 strain, GenBank: GU249598] (amino terminus). Both the VAR2CSA and mSA-VAR2CSA gene fragments were further modified to contain a C-terminal 6xhistidine tag and flanking BamHI and NotI restriction sites used for sub-cloning into the *Baculovirus* vector, pAcGP67A (BD Biosciences). Linearized Bakpak6 *Baculovirus* DNA (BD Biosciences) was co-transfected with pAcGP67A-VAR2CSA or pAcGP67A-mSA-VAR2CSA into Sf9 insect cells for generation of recombinant virus particles. Histidine-tagged recombinant protein was purified on Ni^2+^ sepharose columns from the supernatant of *Baculovirus* infected High-Five insect cells using an ÄKTAxpress purification system (GE-Healthcare).

### Conjugation and purification of mSA-VAR2CSA to L1-Avi VLPs

For production of the VLP-VAR2CSA vaccine, biotinylated HPV16 Avi-L1 VLPs were mixed with 3x molar excess of mSA-VAR2CSA and the sample was incubated at 4°C for 18–24 hours with gentle shaking. Unbound mSA-VAR2CSA was removed by ultracentrifugation over an Optiprep^TM^ step gradient (27%, 33%, 39%) as previously described [22].

The VLP-VAR2CSA vaccine was dialyzed in PBS (0.32 M NaCl, 0.02% PS80) and the relative concentration of VLP-bound mSA-VAR2CSA was estimated by SDS-PAGE using a BSA standard dilution range. Post ultracentrifugation fractions were analyzed by SDS-PAGE or blotted onto a nitrocellulose membrane (GE-Healthcare RPN203E) for detection of L1 or mSA-VAR2CSA (via a 6xhistidine tag) with CamVir-1 (AbD Serotec, Bio-Rad, 7135–2804) or Penta His HRP (QIAGEN, 34460) respectively.

### Immunization of mice

Female C57BL/6 mice (Taconic, Denmark) were immunized by intramuscular injection with 5 μg VLP-coupled mSA-VAR2CSA, soluble mSA-VAR2CSA or soluble naked VAR2CSA on day 0 with no adjuvant. Two booster injections with 2.5 μg of the respective antigens were given on days 21 and 42. Immune-serum was collected on days 14, 35 and 56.

### Anti-VAR2CSA antibody response measured by ELISA

Recombinant VAR2CSA (1 μg/ml in PBS) was coated on Nunc MaxiSorp plates overnight at 4°C. Plates were incubated with blocking buffer for 1 hour at room temperature (RT) to inhibit non-specific binding to the plate. Plates were washed three times in between different steps. Serum samples were diluted in blocking buffer (1:100), added to the wells in three fold dilutions, and incubated for 1 hour at RT. Horseradish peroxidase (HRP)-conjugated polyclonal rabbit anti-mouse Ig (P260 DAKO, Denmark) was diluted 1:3000 in blocking buffer and incubated for 1 hour. Finally, color reactions were developed for 7 min by adding o-phenylenediamine substrate. The HRP enzymatic reaction was stopped by adding 2.5 M H_2_SO_4_ and the optical density was measured at 490 nm using an ELISA plate reader (VersaMax Molecular Devices).

### Parasite culture

Parasites were maintained in culture as described [23]. Briefly, parasites were cultured in 5% hematocrit of human blood (group 0+) in RPMI 1640 (Sigma) supplemented with 0.125 μg/ml Albumax II (Invitrogen) and 2% normal human serum. Atmospheric air was exchanged with a mixture of 1% oxygen and 5% carbon dioxide in nitrogen, whereafter incubation was done at 37°C under static conditions with ad hoc change of culture medium. The FCR3 isolate was selected for binding to CSA by panning on BeWo cells as described [24]. Parasite isolates tested negative for mycoplasma (Lonza) and were regularly genotyped using nested GLURP and MSP-2 primers in a single PCR step, as described [25].

### Inhibition of Binding assays

Parasite DNA was labeled with Tritium by overnight incorporation of titrated hypoxanthine. A 96 well plate (Falcon) was coated with 2 μg/ml of Decorin (Sigma-Aldrich) overnight and blocked with 2% bovine serum albumin (Sigma) as described [23]. Tritium labeled late-stage IEs were MACS purified and added to the 96 well plate in a concentration of 200,000 cells per well. Titrations of serum were added in a total volume of 100 μl in triplicate wells. After incubation for 90 min at 37°C, unbound IEs were washed away by a pipetting robot (Beckman-Coulter). The remaining IEs were harvested onto a filter plate (Perkin-Elmer). After addition of scintillation fluid (Perkin-Elmer) the counts per minute (CPM) recording the number of non-inhibited IE was determined by liquid scintillation counting on a Topcount NXT (Perkin-Elmer). Data were adjusted to percentage of binding by dividing test result with the mean value of wells with IE incubated without serum.

### Ethical Statements

The human blood used for parasite culture was obtained from anonymous donors who provided their informed consent. The animal studies were approved by the Danish Animal Experiments Inspectorate. Approval number: 2013-15-2934-00902/BES.

## Results

### Generation of biotinylated HPV Avi-L1 VLPs

**Fig 1:**
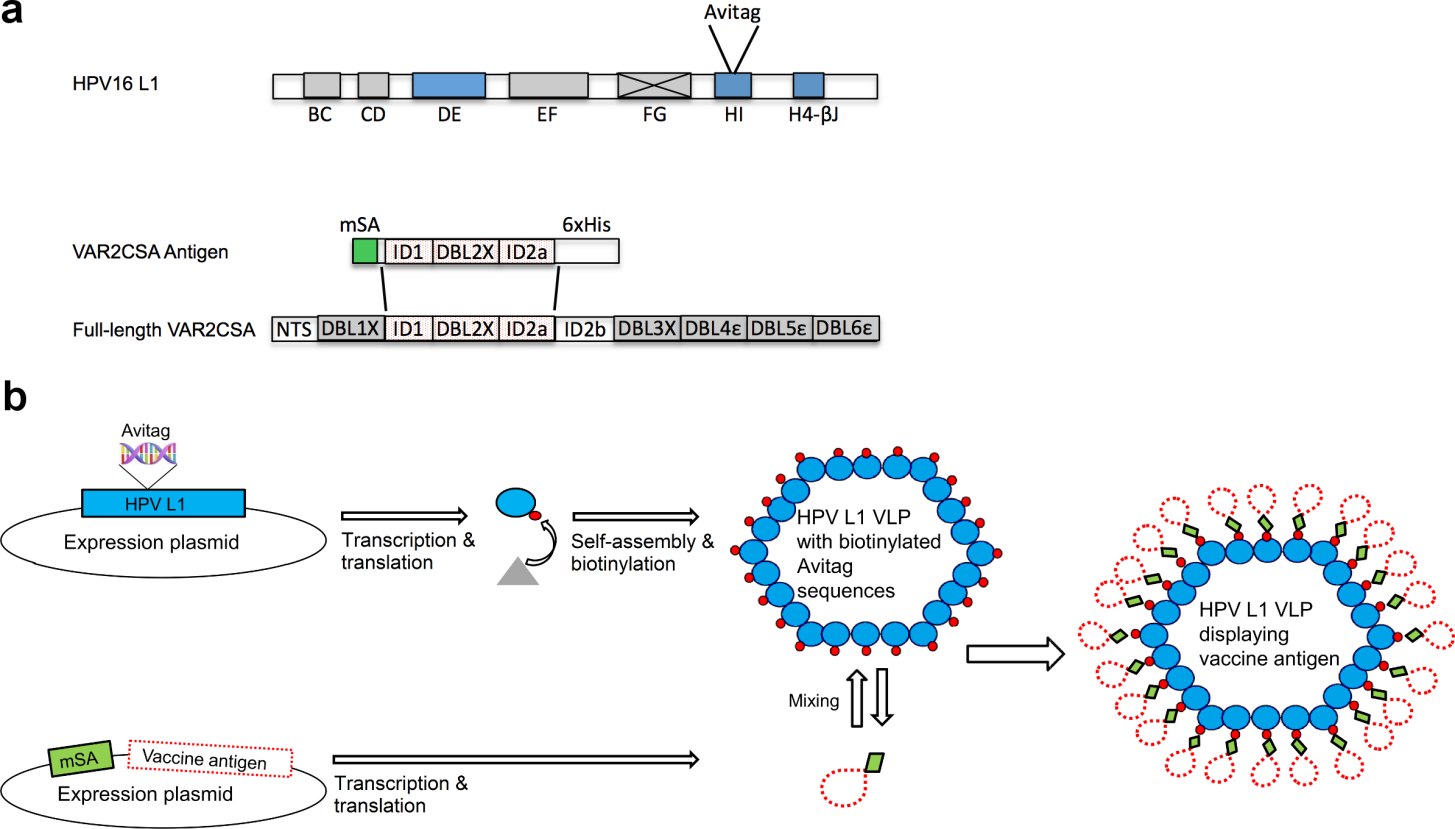
Schematic representation of the VAR2CSA VLP-vaccine components and the assembly system. **a** Structure of HPV16 L1 major capsid protein. The biotin acceptor sequence (AviTag^TM^) was successfully inserted into the protruding DE and HI loops and the H4-βJ coil (blue boxes) of the HPV16 L1. Gray boxes represent regions (loops and coils) of the L1 protein where the AviTag^TM^ could not be inserted (FG loop) or has not been investigated. The VAR2CSA antigen encompasses the extracellular ID1-ID2a (ID1 –Interdomain 1, DBL2X –Duffy binding like 2X and ID2a –Interdomain 2a) domains of the full-length VAR2CSA fused at the N-terminus to a previously described monovalent streptavidin (mSA) [29]. **b** Production process of HPV16 Avi-L1 VLPs displaying consistently oriented mSA-VAR2 antigens in a high-density repetitive manner. The HPV16 Avi-L1 and the mSA-vaccine antigen are separately expressed. The HPV16 Avi-VLPs are subsequently *in vitro* biotinylated (the triangle represents BirA ligase) and finally mixed with the soluble mSA-vaccine antigen. The HPV16 Avi-L1 VLP is theoretically expected to bind one mSA- VAR2CSA antigen per Avi-L1.

**Fig 2:**
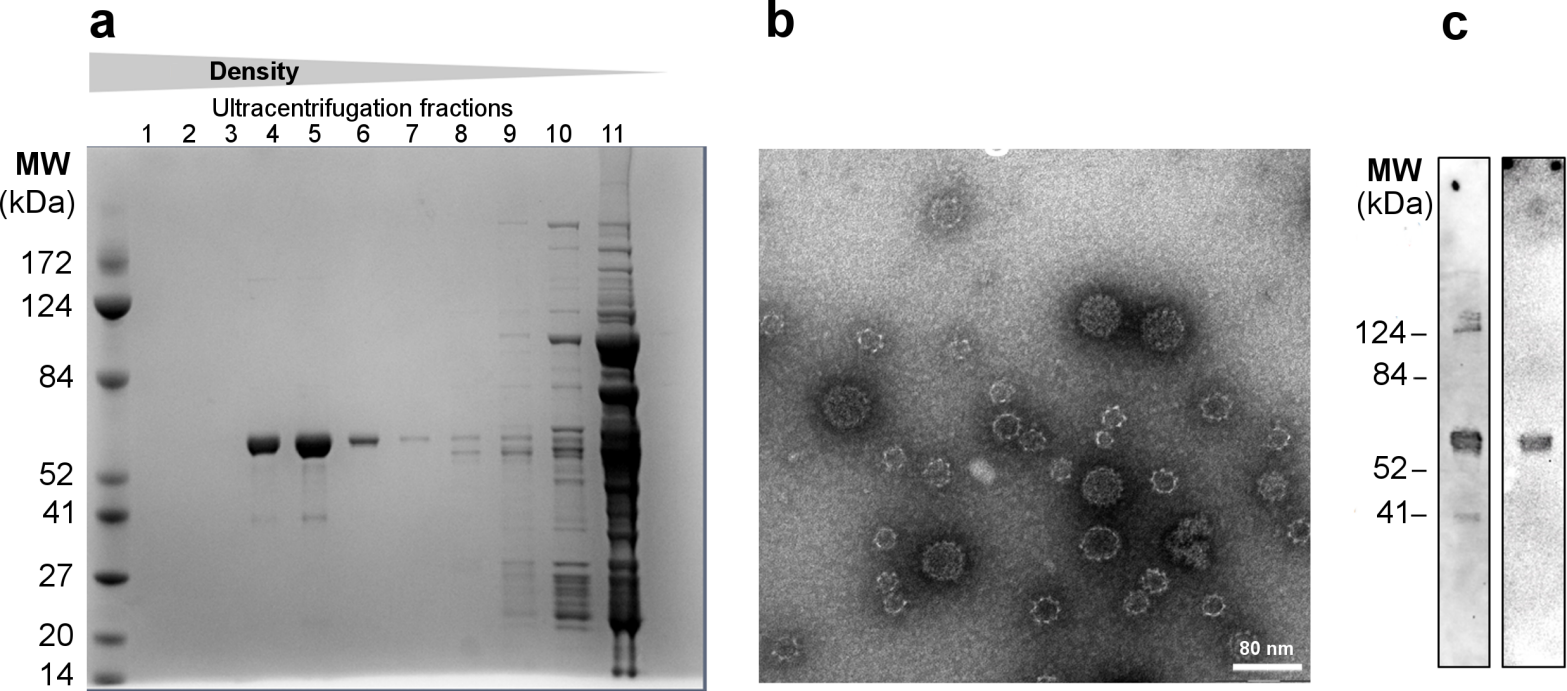
Verification of self-assembly and subsequent *in vitro* biotinylation of HPV16 Avi-L1 VLPs a Purification of HPV16 Avi-L1 VLPs (HI) VLPs was performed by ultracentrifugation (UC) on an iodixanol (Optiprep^TM^) density-gradient (27%/33%/39%). Subsequent reduced SDS-PAGE analyses showed the presence of a 56kDa protein band (theoretical size of Avi-L1) in the high-density UC fractions (4–6) containing particulate material. **b** Transmission-electron microscopy (TEM) analysis of material representing UC fraction 4 post UC purification. To verify the integrity of the chimeric HPV16 Avi-L1 (HI) VLPs, an aliquot of diluted particles was placed on carbon-coated grids, negatively stained with 2% phosphotungstic acid (pH = 7.0) and examined by transmission electron microscopy (TEM) using a CM 100 BioTWIN at magnification x 36,000 (Å), scale bar 80 nm. **c** Western blot analysis of fraction 4 post UC purification. The blot demonstrates the presence of HPV16 Avi-L1 (56kDa) detected by Camvir-1 (lane one) and successful biotinylation of HPV16 Avi-L1 using Strep-HRP to detect biotin (lane two).

The biotin acceptor site (AviTag^TM^) sequence (GLNDIFEAQKIEWHE) was genetically inserted at four different positions in the HPV16 L1 sequence, which based on the crystal structure of HPV16 L1 capsid protein, correspond to surface exposed loops in the assembled VLP structure [26]. The rationale for choosing these positions was to allow VLP assembly while at the same time exposing the AviTag^TM^ sequence for subsequent biotinylation and coupling of mSA-VAR2CSA (Fig 1**A**). The chimeric Avi-L1 constructs were expressed in High-Five cells and allowed to assemble into VLPs (Fig 1**B**). Formation of VLPs was confirmed by performing density gradient ultracentrifugation followed by SDS-PAGE analysis. This analysis indicated that three of the recombinant proteins, Avi-L1 (HI), Avi-L1 (DE) and Avi-L1 (H4-βJ coil) formed VLPs as the majority of the expressed protein were present in high molecular/density fractions 4–6 after ultracentrifugation (Fig 2**A**) [22]. The Avi-L1 (FG) protein was exclusively found in the low density fractions containing non-particulate soluble protein, indicating that the AviTag^TM^ insertion, in this case, prevented VLP assembly. The identity of Avi-L1 proteins in the high-density fractions was confirmed by western blot analysis using a monoclonal anti-HPV16 L1 antibody (Fig 2**C**). VLP assembly of the Avi-L1 constructs was verified by transmission electron microscopy (TEM) showing a heterogeneous population of VLPs of different sizes (28–60nm) (Fig 2**B**), representing different icosahedral assembly symmetries of which roughly 30% had the size/appearance of native HPV16 L1 VLPs [27]. The particles were subsequently biotinylated (Fig 2**C**) and analyzed by TEM.

### Construction of the displayed antigen VAR2CSA

**Fig 3:**
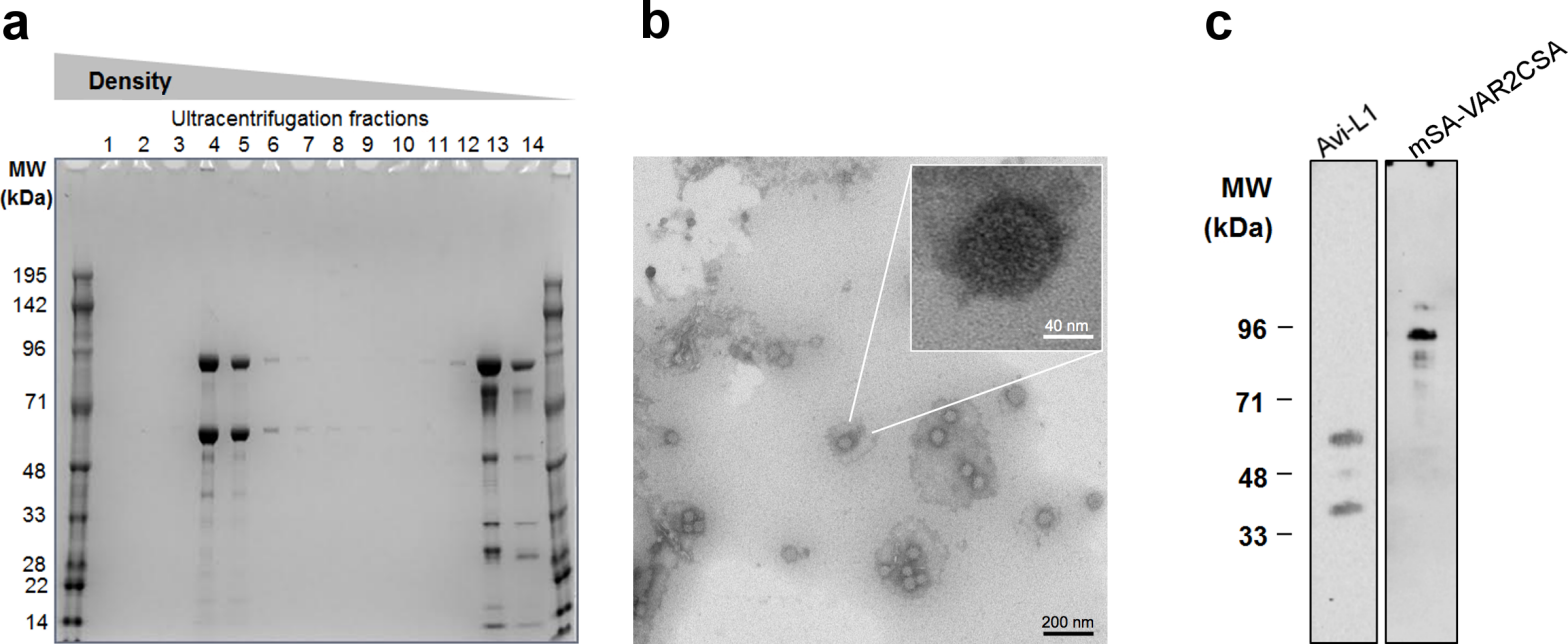
HPV16 Avi-L1 VLP coupled to mSA-ID1-ID2a analyzed by ultracentrifugation followed by SDS-PAGE, Western blot and TEM analysis. **a** After coupling of the mSA-ID1-ID2a antigen to the HPV16 Avi-L1 VLPs, excess antigen was removed by UC over an Optiprep^TM^ gradient (27%/33%/39%). Reducing SDS-PAGE analysis showed the presence of two protein bands corresponding to the size of HPV16 Avi-L1 (56kDa) and mSA-VAR2CSA (85kDa), respectively, in the high-density fractions (4–6) post UC purification. Excess unbound mSA-VAR2CSA was present in the higher UC fractions (12–14) containing soluble proteins. **b** Transmission electron microscopy (TEM) analysis of material from UC fraction 4 containing HPV16 Avi-L1 VLPs coupled with mSA-VAR2CSA. An aliquot of diluted particles was placed on carbon-coated grids, negatively stained with 2% phosphotungstic acid (pH = 7.0) and examined by transmission electron microscopy (TEM) using a CM 100 BioTWIN at magnification x 36,000 (Å). Black scale bar 200 nm, enhanced section white scale bar 40 nm. **c** Western blot analysis of fraction 4 post UC purification of mixed HPV16 Avi-L1 and mSA-VAR2CSA. The blot confirms the presence of HPV16 Avi-L1 and mSA-VAR2CSA detected by Camvir-1 and α-PENTA HIS-tag, respectively.

The shortest truncated VAR2CSA polypeptide sequence that is able to bind CSA covers the ID1-ID2a region of the full-length protein (Fig 1**A**) [8,28]. This construct (herein referred to as VAR2CSA) was genetically fused at the amino terminus to monomeric streptavidin (mSA) which can bind biotin with high affinity [29,30]. To avoid VLP aggregation we used a monomeric form of streptavidin which is advantageous to native streptavidin as the latter contains four biotin binding sites [31]. The chimeric construct expressed high levels of soluble protein and retained a structure capable of binding CSA (data not shown). Purified mSA-VAR2CSA was subsequently mixed with the biotinylated VLP (1:3 HPV L1/antigen), and examined by ultracentrifugation followed by SDS-PAGE and western blot analysis. High-density ultracentrifugation fractions (4–5) contained both mSA-VAR2CSA and Avi-L1 protein, which was estimated by densitometric analysis to be present at a 0.6:1 molar ratio. Excess mSA-VAR2CSA was present in the low-density fractions (12–14) (Fig 3**A** and 3**C**). The co-localization of Avi-L1 and mSA-VAR2CSA, indicated that the mSA-VAR2CSA antigen was bound to the surface of Avi-L1 VLPs at high density and that these large protein complexes had been separated from the excess soluble mSA-VAR2CSA (Fig 3**A**). To confirm that co-localization of mSA-VAR2CSA and Avi-L1 VLPs was caused by the specific interaction between mSA and biotin, the procedure was repeated using unbiotinylated VLPs. This control experiment showed that ultracentrifugation efficiently separated soluble mSA-VAR2CSA from unbiotinylated VLPs (S1 Fig).

Antigen-coupled VLPs were further examined by TEM, showing particles of a comparably larger size (~70 nm) than the non-coupled VLPs (~30–60 nm) (Fig 3**B**). This observation was further examined by dynamic light scattering (DLS) analysis, which confirmed that mixing of mSA-VAR2CSA with biotinylated Avi-L1 VLPs resulted in measurably larger particles with an average diameter of ~70 nm (12.9% Pd) compared to naked Avi-L1 VLPs (≤60 nm, 23.9% Pd). Importantly, this analysis also confirmed that such large complexes were not formed after mixing unbiotinylated VLPs with mSA-VAR2CSA, demonstrating that mSA-VAR2CSA is, in fact, bound to the surface of Avi-L1 VLPs via the specific affinity interaction between mSA and biotin.

Together, these results indicate that the produced mSA-VAR2CSA VLP vaccine consists of non-aggregated HPV16 Avi-L1 VLPs displaying dense, repetitive arrays of the mSA-VAR2CSA antigen presented in a consistent orientation, as illustrated schematically in Fig 1**B**.

### The possibility of inserting AviTag^TM^ into other coat proteins forming papilloma VLP

**Fig 4:**
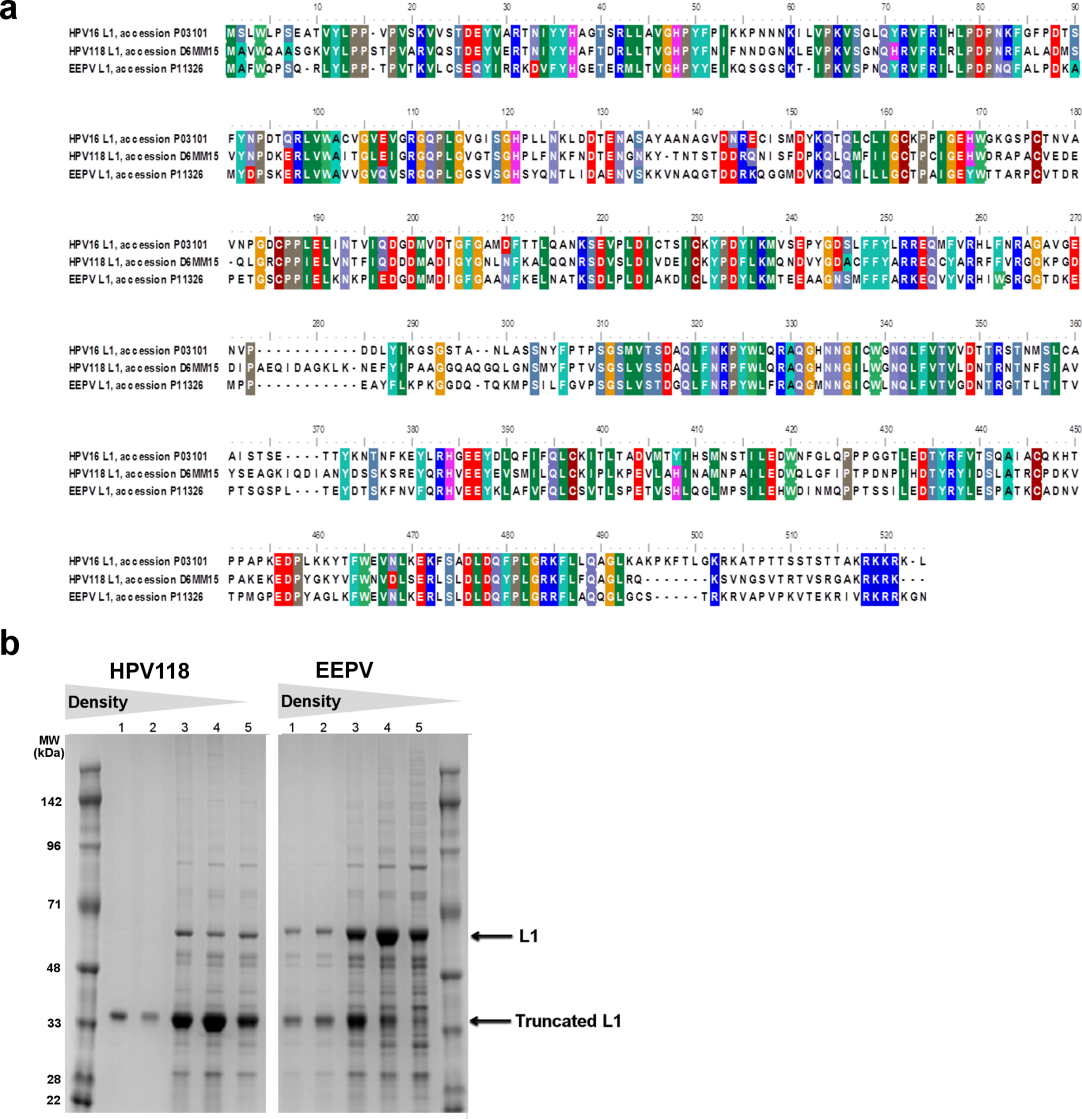
Other PV VLPs with AviTag^TM^ inserted in DE-loop. The HPV16 L1 VLP was used as VLP platform for proof of concept in this study. However, the AviTag^TM^ can also be inserted into the DE loop of the major capsid protein from other papilloma viruses while retaining the ability to self-assemble into VLPs, as demonstrated in this figure. **a** Multiple sequence alignment of the HPV16 L1, HPV118 L1 and major capsid protein from European Elk papilloma virus (PAPVE). **b** Purification of HPV118 Avi-L1 and PAPVE Avi-L1 VLPs were performed by UC over an Optiprep^TM^ density gradient (27%/33%/39). Subsequent reduced SDS-PAGE analysis of high-density UC fractions (3–5) show the presence of a protein band of 56 kDa corresponding to the full-length Avi-L1 protein. These fractions also contain an intense protein band of approximately 43kDa, which may represent a truncated Avi-L1 product.

The HPV16 L1 genotype, used as VLP platform in this study, constitutes one of the most prevalent oncogenic HPV types [32]. This genotype is included in all licensed HPV vaccines, which are being administered to millions of young women every year. Thus, the immunogenicity of this VLP platform could be impeded by pre-existing immunity to towards the HPV L1 major capsid protein. We therefore examined whether insertion of the AviTag^TM^ sequence was compatible with VLP formation in other HPV genotypes as well as in non-human PV. The AviTag^TM^ sequence was inserted in the L1 major capsid protein of the European elk PV as well as in HPV genotype 118 at a position corresponding to the fusion site in the HPV16 Avi-L1 (DE) (Fig 4**A**). The two protein sequences were expressed and purified as previously described. For both constructs a L1 band of the expected protein size (56kDa) was found in the high-density ultracentrifugation fractions although the majority of protein present in these fractions was of a lower molecular size, possibly representing a L1 truncation (Fig 4**B**). These data suggest that insertion of the AviTag^TM^ sequence into other PV types is a feasible strategy for avoiding the potential issue of pre-existing immunity.

### Immunogenicity of the mSA-VAR2CSA VLP vaccine in C57BL/6 mice

**Fig 5:**
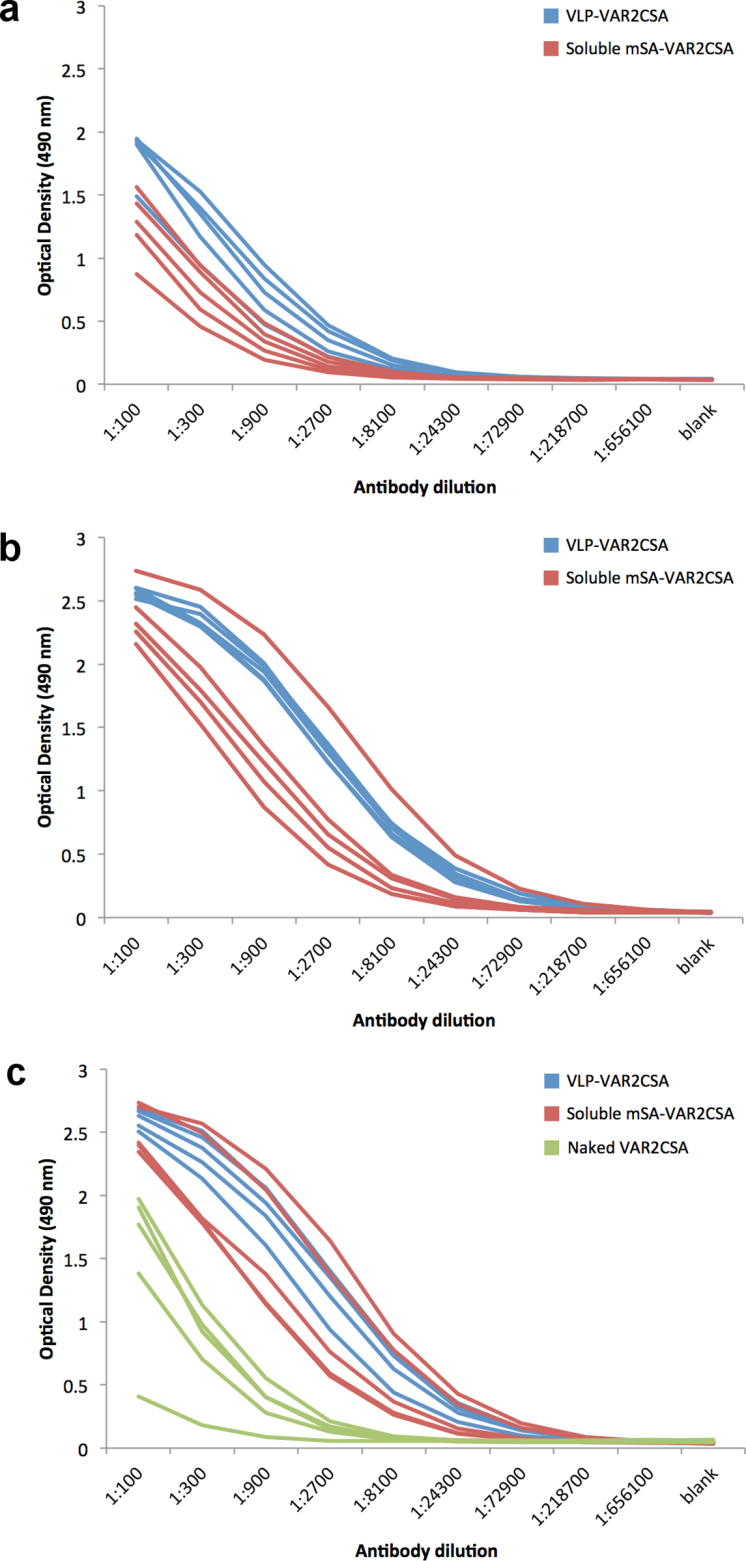
Reactivity of sera from vaccinated mice by ELISA. C57BL/6 mice were immunized three times with three-week intervals. Serum samples were collected two weeks after each immunization. Total VAR2CSA-specific immunoglobulin was measured in a serial dilution of mouse anti-sera by ELISA using recombinant VAR2CSA as the solid phase capturing antigen. HRP conjugated anti-mouse Ig antibodies were used for detection by measuring absorbance at OD 490 nm. Serum reactivity from individual mice vaccinated with mSA-VAR2CSA coupled HPV16 Avi-L1 (HI) VLPs (blue) or uncoupled mSA-VAR2CSA (red) are shown after first (**a**), second (**b**) and third (**c**) immunization where each line shows the reactivity of one animal. Green curves represent sera from mice vaccinated with soluble naked VAR2CSA and is a pool of sera obtained after 2^nd^ and 3^rd^ bleed.

The immunogenicity of the mSA-VAR2CSA VLP vaccine was tested in C57BL/6 mice vaccinated three times with three week intervals. ELISA was used to measure total immunoglobulin (Ig) levels against VAR2CSA in sera obtained from mice immunized with mSA-VAR2CSA VLP, soluble mSA-VAR2CSA or soluble naked VAR2CSA (Fig 5). After three immunizations the VAR2CSA specific Ig levels were higher in sera from mice immunized with the mSA-VAR2CSA Avi-L1 VLP vaccine than in sera from mice immunized with soluble naked VAR2CSA (Fig 5**C**). After 1^st^ and 2^nd^ immunization sera from mSA-VAR2CSA Avi-L1 VLP immunized mice had statistically significantly higher Ig endpoint titers compared with sera from mice immunized with the soluble mSA-VAR2CSA vaccine (*P* = 0.014 and *P* = 0.018, respectively). This difference was, however, not statistically significant after the 3^rd^ immunization (*P* = 0.058) (Table 1) where both vaccines seem to have reached a similar plateau.

**Table 1:** Serum endpoint titers (median {25 and 75 percentiles}) obtained with the different immunogens.

	After first immunization	After second immunization	After third immunization
**1. Soluble VAR2CSA**	Not done	Not done	8,100^a^{8,100:8,100}
**2. Soluble mSA-VAR2CSA**	24,300^a^ {24,300:24,300}	218,700^a^ {72,900:218,700}	218,700^a^ {72,900:218,700}
**3. mSA-VAR2CSA Avi-L1 VLP**	72,900^a^ {72,900:218,700}	656,100^a^ {656,100:656,100}	218,700^a^ {218,700:656,100}
**P-Value** ^**b**^	0.014 (2. vs 3.)	0.018 (2. vs 3.)	0.0072 (1. vs 2.)0.0072 (1. vs 3.) 0.058 (2. vs 3.)

^a^ Endpoint titer, defined as the reciprocal of the highest serum dilution giving an OD measurement above the cutoff. The cutoff was set to be three standard deviations above the mean negative control reading.

^b^ *P* values were calculated using Wilcoxon rank sum test.

### Functionality of the mSA-VAR2CSA VLP vaccine induced anti-VAR2 antibodies

**Fig 6:**
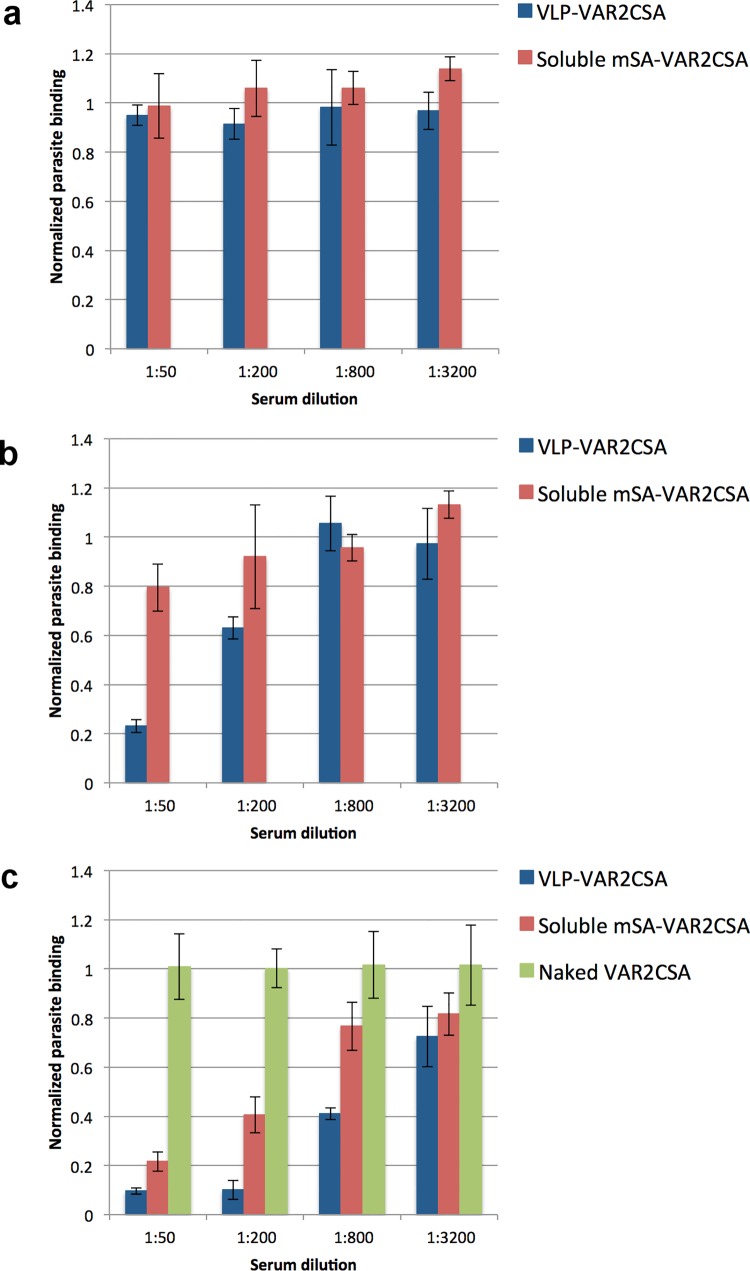
Display of VAR2CSA on HPV16 L1-AviTag VLPs assessed by parasite inhibition assay. The functional antibody response was assessed by measuring the capacity of mouse anti-sera to inhibit binding between native VAR2CSA expressed on parasitized erythrocytes and CSA in a static binding-assay. *P*. *falciparum* (FCR3 genotype)-infected red blood cells, expressing the native VAR2CSA, were first incubated with mouse anti-serum (4 fold dilution series, starting from 1:50) and then allowed to incubate on decorin coated plates for 90 min. Unbound IE were washed away and the remaining IEs were quantified. Normalized parasite binding after incubation with pooled anti-sera from mice (n = 5) vaccinated with mSA-VAR2CSA-coupled HPV16 Avi-L1 VLPs (blue) or soluble mSA-VAR2CSA (red) are shown after first (**a**), second (**b**) and third (**c**) immunization. The green piles in Fig 6**c** represent anti-sera from mice vaccinated with soluble naked VAR2CSA and is a pool of sera from 2^nd^ and 3^rd^ bleed.

Antisera were examined for their ability to block the binding between native VAR2CSA expressed on the surface of parasite-infected erythrocytes and immobilized CSA. After first immunization, none of the three vaccines had induced efficient levels of functional binding-inhibitory antibodies, leading to full binding of parasites (Fig 6**A**). However, after the second round of immunizations 1:50 diluted serum from mSA-VAR2CSA VLP immunized mice inhibited the binding between IE and CSA by approximately 70%. In comparison, the soluble mSA-VAR2CSA vaccine only inhibited approximately 20%, while no inhibition was seen for the soluble naked VAR2CSA vaccine (Fig 6**B**). After three immunizations, 1:200 diluted serum from mice immunized with the mSA-VAR2CSA VLP vaccine showed roughly 90% binding-inhibition, while the sera from mice immunized with the soluble mSA-VAR2CSA vaccine inhibited parasite binding by approximately 60%. By contrast, the soluble naked VAR2CSA vaccine failed to induce any binding-inhibitory antibodies (Fig 6**C**).

## Discussion

Placental malaria constitutes a large problem in malaria endemic countries and current control strategies based on the administration of repeated curative doses of antimalarial treatment (intermittent preventive treatment during pregnancy, IPTp) during the 2^nd^ and 3^rd^ trimester are compromised by development of parasite drug resistance and a general low effective delivery of IPTp programme in sub-Saharan African countries. IPTp also suffers from the inherent problem that the first treatment dose is often given after the initial exposure to malaria and irreversible damage to the placenta has already occurred [33]. An effective vaccine protecting women against PM infection would circumvent these problems. For such a vaccine to be practical it must be administered prior to conception. Most realistically the target group would be pre-puberty girls and therefore several years are likely to lapse between vaccination and exposure. Consequently, vaccine efficacy would have to be retained over several years; a requirement that soluble protein-based vaccines have generally proven unable to meet. VLP-based HPV vaccines, however, have been shown to induce high antibody titer responses, even after a single dose, which is attributed to the high-density repetitive display of the neutralizing epitopes. Furthermore, combined evidence indicates that soluble proteins may become very potent immunogens, provided that they are presented to the immune system in a similar virus-like display [15,18]. Many are working to improve the VLP display challenges e.g. the recent approach presented in the study by Koho T. *et al*. [34]. Genetic fusion of small peptide sequences in the viral structural proteins have so far been the most successful strategy to obtain VLP-display of heterologous antigenic epitopes whereas cross-linking chemistry has been employed to facilitate coupling of more complex antigens to pre-assembled VLPs. However, in the case of the PM vaccine candidate, VAR2CSA, neither of the conventional approaches is optimal *i*.*e*. genetic fusion is not possible because important neutralizing epitopes remain to be identified. Furthermore, the presence of multiple surface-reactive cysteine residues hampers the ability to site-specifically couple the complex VAR2CSA antigen to pre-assembled VLPs using chemical cross-linking. Here, we describe the development and evaluation of a novel VLP-display platform based on the highly immunogenic HPV16 L1 VLP, which in this study is used as a scaffold for displaying the 66kDa VAR2CSA antigen. We show that immunization of C57BL/6 mice with the mSA-VAR2CSA Avi-L1 VLP vaccine results in higher levels of VAR2CSA-specific Ig compared to when immunizing with the naked soluble VAR2CSA protein. A parasite binding-inhibition assay furthermore confirmed that the VLP-based vaccine was markedly superior at inducing functional antibodies compared to the soluble protein-based vaccine containing naked soluble VAR2CSA. The VAR2CSA antigen has previously been shown to induce functional antibodies when adsorbed to aluminum hydroxide [35] and Freund’s complete adjuvant [23]. In the current experimental setup we thus used a relatively low antigen dose and did not use extrinsic adjuvant, in order not to mask a potential VLP-display effect. It thus remains to be investigated if adding *e*.*g*. aluminum hydroxide could enhance the immunogenicity of the mSA-VAR2CSA VLP vaccine even further and, importantly, how this vaccine would perform against a similarly adjuvanted soluble protein-based vaccine. Further long-term studies are moreover needed to clarify the effect the increased immunogenicity of the mSA-VAR2CSA VLP vaccine has on the durability of the induced immune response.

It is intriguing that the soluble mSA-VAR2CSA vaccine performed better than soluble naked VAR2CSA vaccine. It could be hypothesized that the improved immunogenicity could be caused by the presence of a T-cell epitope in the mSA domain [31]. Alternatively, the affinity for biotin could affect the immune response via unknown mechanisms. Although the VLP-based vaccine showed a tendency of inducing a faster, slightly more potent (based on higher antibody titres after 1^st^ and 2^nd^ immunization) and more effective (based on capacity of inhibiting parasite binding to CSA) immune response compared to the soluble protein mSA-VAR2CSA vaccine, this difference was not great.

The fact that the mSA-VAR2CSA VLP vaccine is built on the HPV L1 VLP platform suggests that it could be modified and used as a combinatorial vaccine to protect women against both human papillomavirus and PM. Such a dual vaccine would have the same target population (*i*.*e*. females of pre-reproductive age) and could be tailored to make a cost-effective vaccine specifically targeted at women in African countries. We have data indicating that the Avitag^TM^ can be incorporated into L1 of other PV types (e.g. HPV118 and Elk PV), and our strategy could thus likely be used to target HPV serotypes, which are prevalent in Africa, but not covered by the licensed HPV vaccines.

Finally, our data serve as proof of principle for how to deliver other complex vaccine antigens to the immune system on a HPV VLP platform. Compared to existing strategies for coupling larger polypeptides to pre-assembled VLPs (e.g. by using hetero bi-functional cross-linkers to bridge exposed cysteines on the antigen to lysine residues on the of the VLP surface [36] our technology ensures that the antigen is coupled in a consistent orientation while having the potential to present the antigen at high density (1:1 antigen/ HPV L1) on the VLP surface (Fig 2**A** and 2**B**), together leading to an optimal display of epitopes. The described technology simply requires that the vaccine antigen can be expressed as a fusion partner with the mSA while retaining important neutralizing epitopes. Recombinant soluble VAR2CSA is, compared to many other proteins, very immunogenic when adsorbed to aluminum hydroxide and can induce high antibody titers in mice upon injection of very low doses (unpublished data). The present study may thus not reveal the full potential of the described technology and we thus propose that this new VLP platform, advantageously, can be used to enhance immune responses against other vaccine antigens of low immunogenicity or to break immune tolerance against self-antigens.

In summary, we have demonstrated a high-density coupling of mSA-VAR2CSA to the surface of biotinylated HPV16 Avi-L1 VLPs and have showed that the resulting VLP-based vaccine was effective in inducing functional antibodies capable of inhibiting binding between IE and CSA *in vitro*. The described technology may serve as a VLP-display platform to improve immunogenicity of other vaccine antigens.

## Supporting Information

S1 Fig mSA-VAR2CSA does not co-localize with unbiotinylated VLPs. (TIF)
