# Supplementary figures and images for: A Novel Virus-Like Particle Based Vaccine Platform Displaying the Placental Malaria Antigen VAR2CSA
Source: PLoS One. 2015 Nov 23;10(11):e0143071. doi: 10.1371/journal.pone.0143071 (PMC4657905; doi:10.1371/journal.pone.0143071)

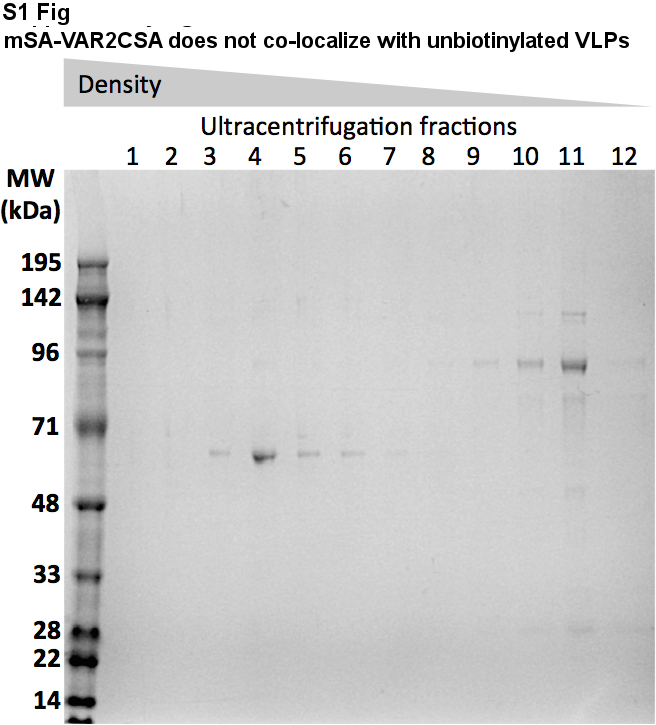

Supplement: S1 Fig — (TIF) [file pone.0143071.s001.tif]
